# Efficacy and safety of glucosamine, diacerein, and NSAIDs in osteoarthritis knee: a systematic review and network meta-analysis

**DOI:** 10.1186/s40001-015-0115-7

**Published:** 2015-03-13

**Authors:** Jatupon Kongtharvonskul, Thunyarat Anothaisintawee, Mark McEvoy, John Attia, Patarawan Woratanarat, Ammarin Thakkinstian

**Affiliations:** Section for Clinical Epidemiology and Biostatistics, Faculty of Medicine, Faculty of Medicine Ramathibodi Hospital, Ratchathewi, Chang Wat Bangkok, Bangkok, 10400 Thailand; Centre for Clinical Epidemiology and Biostatistics, The University of Newcastle, University Drive, Newcastle, NSW 2308 Australia; Centre for Clinical Epidemiology and Biostatistics, School of Medicine and Public Health, Hunter Medical Research Institute, University of Newcastle, University Drive, Newcastle, NSW 2308 Australia; Department of Orthopaedics, Faculty of Medicine Ramathibodi Hospital, Bangkok, Mahidol University, Highway 3310, Bangkok, 73170 Thailand

**Keywords:** Osteoarthritis, Gonarthrosis, Systematic review, Network meta-analysis, SYSADOA

## Abstract

**Background:**

To conduct a systematic review and network meta-analysis of randomized controlled trials (RCTs) with the aims of comparing relevant clinical outcomes (that is, visual analog scores (VAS), total and sub-Western Ontario and McMaster Universities Osteoarthritis index (WOMAC) scores, Lequesne algofunctional index, joint space width change, and adverse events) between diacerein, glucosamine, and placebo.

**Methods:**

Medline and Scopus databases were searched from inception to 29 August 2014, using PubMed and Scopus search engines and included RCTs or quasi-experimental designs comparing clinical outcomes between treatments. Data were extracted from original studies. A network meta-analysis was performed by applying weight regression for continuous outcomes and a mixed-effect Poisson regression for dichotomous outcomes.

**Results:**

Thirty-one of 505 identified studies were eligible. Compared to placebo, glucosamine showed a significant improvement with unstandardized mean differences (UMD) in total WOMAC, pain WOMAC, function WOMAC, and Lequesne score of −2.49 (95% confidence interval (CI) −4.14, −0.83), −0.75 (95% CI: −1.18, −0.32), −4.78 (95% CI: −5.96, −3.59), and −1.03 (95% CI: −1.34, −0.72), respectively. Diacerein clinically improves visual analog scores, function WOMAC, and stiffness WOMAC with UMD values of −2.23 (95% CI: −2.82, −1.64), −6.64 (95% CI: −10.50, −2.78), and −0.68 (95% CI: −1.20, −0.16) when compared to placebo.

**Conclusions:**

The network meta-analysis suggests that diacerein and glucosamine are equally efficacious for symptom relief in knee OA, but that the former has more side effects.

**Electronic supplementary material:**

The online version of this article (doi:10.1186/s40001-015-0115-7) contains supplementary material, which is available to authorized users.

## Background

Osteoarthritis (OA) is the most common chronic joint disease of the older patient. The primarily affected joints are the knee and hip. The progression of the disease is influential on quality of life. This included functional and social activities, body image, and emotional well-being. In non-operative treatment, pain reduction and improved function are the primary goals. Management of mild degree OA of the knee mainly consists of medical treatment and lifestyle modifications. Non-steroidal anti-inflammatory drugs (NSAIDs) are the most commonly prescribed agents for pain management, but they increase the risk of gastrointestinal (GI) bleeding and vascular adverse events [1,2]. Therefore, second-line drugs such as symptomatic slow-acting drugs for OA (SYSADOA) which include glucosamine sulfate, glucosamine hydrochloride, chondroitin sulfate, hyaluronic acid, avocado soybean unsaponifiables (ASU), and diacerein are more commonly used. These drugs may improve patient symptoms as well as reduce cartilage degradation [3,4], also having decreased occurrence of GI adverse events when compared to NSAIDs. Two drugs are recommended by the European League Against Rheumatism guidelines 2003. These include an interleukin-1 (IL-1B) inhibitor (diacerein) and glucosamine. However, these drugs have a slow onset and a prolonged residual effect. The diacerein and glucosamine groups have the greatest amount of randomized controlled trial (RCT) studies and meta-analysis when compared to all other SYSADOA. The results of all the studies show that diacerein and glucosamine improve symptoms and decrease structural progression in OA of the knee when compared to NSAIDs and placebo. Previous systematic reviews [3-6] have shown that diacerein had higher efficacy in reducing pain and Lequesne index, but increased risk of diarrhea when compared to placebo [4,6]. Similar effects were observed in systematic reviews of the efficacy of glucosamine, which showed a significant reduction in pain when compared to placebo but no effect on minimal joint space narrowing or adverse events [3,5]. However, no RCTs directly compared the clinical efficacy and safety of diacerein with glucosamine. We therefore conducted a systematic review with a network meta-analysis of RCTs with the aim of comparing relevant clinical outcomes (that is, visual analog score, Western Ontario and McMaster Universities Osteoarthritis index (WOMAC) score, Lequesne algofunctional index, joint space width change, and adverse events) between diacerein, glucosamine, NSAIDs, and placebo. The main outcomes that were focused on in this study included pain, functional assessment, joint space width change, and safety issues of the medications.

## Methods

### Search strategy

The Medline and Scopus databases were used to identify relevant studies published in English from the date of inception to 29 August 2014. The PubMed and Scopus search engines were used to locate studies using the following search terms: (osteoarthritis, degenerative arthritis, adult, older person), (symptomatic slow acting drug for osteoarthritis; SYSADOA, diacerein, glucosamine), (pain, function, score, grade, WOMAC, Knee Society Score (KSS), motion, radiographic grading, X-ray, MRI, Kellgren-Lawrence), (clinical trial, RCT, randomized controlled trial). Search strategies for Medline and Scopus are described in Additional file 1. Relevant studies from the reference lists of identified studies and previous systematic reviews were also explored.

### Selection of studies

**Figure 1:**
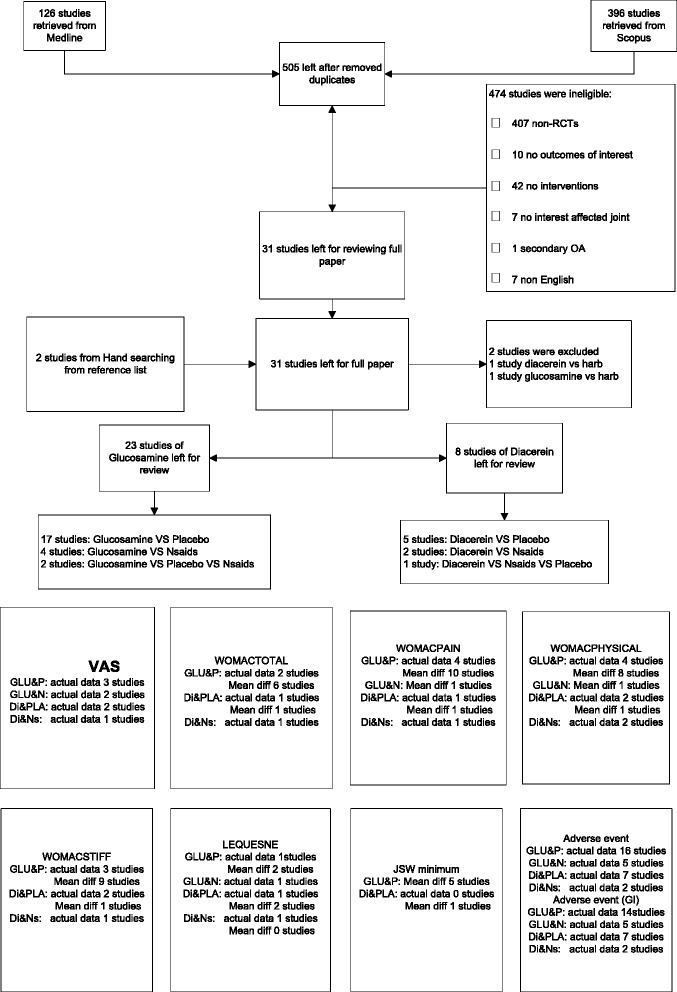
**Flow of study selection.**

Identified studies were selected by one author (J.K.) and randomly checked by A.T. Titles and abstracts were initially screened; full papers were then retrieved if a decision could not be made from the abstracts. The reasons for ineligibility or exclusion of studies were recorded and described (Figure 1).

### Inclusion criteria

Randomized controlled trials or quasi-experimental designs comparing clinical outcomes between treatments in primary OA patients’ knee were eligible if they met the following criteria:

- Compared clinical outcomes between glucosamine (either glucosamine sulfate or glucosamine hydrochloride) and diacerein, or each of these treatments with other comparators (for example, placebo, non-steroidal anti-inflammatory drugs).
- Compared at least one of the following outcomes: pain score, function, patient/physician global assessments, range of motion, joint space width difference, and adverse events.
- Had sufficient data to extract and pool: reported mean, standard deviation (SD), numbers of subjects according to treatments for continuous outcomes, and number of patients according to treatment for dichotomous outcomes.

### Data extraction

Two reviewers (J.K. and T.A.) independently performed data extraction using standardized data extraction forms. General characteristics of the study (mean age, gender, body mass index, duration of OA, pain score, and functional scores at baseline) were extracted. The number of subjects, mean, and SD of continuous outcomes (pain by visual analog score (VAS), total and sub-WOMAC scores, and Lequesne algofunctional index) between the groups were extracted. Cross-tabulated frequencies between treatments and adverse events were also extracted. Any disagreements were resolved by discussion and consensus with a third party (A.T.).

### Risk of bias assessment

Two authors (J.K. and T.A.) independently assessed risk of bias for each study. Six study quality domains were considered, including sequence generation, allocation concealment, blinding (participant, personnel, and outcome assessors), incomplete outcome data, selective outcome reporting, and other sources of bias [7]. Disagreements between two authors were resolved by consensus and discussion with a third party (A.T.).

### Outcomes

The outcome of interests were pain VAS, total and sub-WOMAC scores (pain, stiffness, and function), Lequesne algofunctional index, joint space width (minimum), and adverse events. Methods of measure for these outcomes were used according to the original studies. Briefly, this includes the VAS pain scale from 0 to 10; the WOMAC score that consists of pain (0 to 20), stiffness (0 to 8), and function (0 to 68) with total scores of 0 to 96 [8]. The Lequesne algofunctional index measured pain (0 to 10), maximum distance walked (0 to 6), and activities of daily living (0 to 8) with total scores of 0 to 24 [9,8]. For joint space width change, lower values of these scores refer to better outcomes. Adverse events were considered as composite and separate outcomes of the following: a musculoskeletal disorder, respiratory disorder, genitourinary tract disorder or central nervous system disorder, and GI adverse effects.

### Statistical analysis

Direct comparisons of continuous outcomes were measured at the end of each study between glucosamine *versus* placebo and diacerein *versus* placebo and were then pooled using an unstandardized mean difference (UMD). Heterogeneity of the mean difference across studies was checked using the *Q* statistic, and the degree was quantified using the *I*^2^ statistic. If heterogeneity was present (*P* value <0.10 or the *I*^2^ > 25%), the UMD was estimated using a random effects model; otherwise, a fixed-effects model was applied.

For dichotomous outcomes, a relative risk (RR) of adverse reactions of treatment comparisons at the end of each study was estimated and pooled. Heterogeneity was assessed using the same method as mentioned previously. If heterogeneity was present, the DerSimonian and Laird method [10] was applied for pooling; otherwise, the fixed-effects model by inverse variance method was applied. Meta-regression was applied to explore the source of heterogeneity (for example, mean age, percentage of females, bone mass index (BMI), Kellgren-Lawrence grading, duration of OA) if data was available. Publication bias was assessed using contour-enhanced funnel plots [11,12] and Egger tests [13].

For indirect comparisons, network meta-analyses were applied to assess all possible effects of treatment measured at different times if summary data were available for pooling [14-16]. A linear regression model weighted by inverse variance was applied to assess the treatment effects with adjustment for study effects and time for continuous outcomes. For adverse events, a mixed-effect Poisson regression was applied to assess treatment effects [15]. Summary data was expanded to individual patient data using the “expand” command in STATA. Treatment was considered as a fixed-effect, whereas the study variable was considered as a random-effect in a mixed-effect model. The pooled RR and its 95% confidence intervals (CIs) were estimated by exponential coefficients of treatments. All analyses were performed using STATA version 12.0 [17]. A *P* value <0.05 was considered statistically significant, except for the test of heterogeneity where *P* value < 0.10 was used.

## Results

Among 505 identified studies and 2 referred studies, 31 studies [18-48] were eligible for data extraction. Reasons for ineligibility are described in Figure 1. Characteristics of the 31 studies [18-44,46-48,45] are described in Table 1.

**Table 1:** **Characteristics of included studies**

**Author**	**Years**	**Follow-up**	**Intervention (mg)**	**Comparator**	**Age**	**Female (%)**	**BMI**	**Duration**	**Outcome**
Pujalte JM	1980	8 weeks	GS (1,500)	Placebo	61.7	85	-	-	VAS, AR
Lopes VA	1982	8 weeks	GS (1,500)	NSAIDs	56.4	74	-	3.2	VAS, AR
Muller FH	1994	4 weeks	GS (1,500)	NSAIDs	54	42.3	-	4.8	Lequesne, AR
Noack W	1994	4 weeks	GS (1,500)	Placebo	55	60.3	-	-	Lequesne, AR
Nguyen M	1994	8 weeks	D (50)	NSAIDs, placebo	62	62.7	-	5.25	VAS, Lequesne, AR
Qiu GX	1998	4 weeks	GS (1,500)	NSAIDs	56.4	79	-	-	VAS, AR
Houpt JB	1999	8 weeks	GH (1,500)	Placebo	64.5	64.4	-	8.3	WOMAC, AR
Rindone JP	2000	4, 8 weeks	GS (1,500)	Placebo	63.5	5.1	-	13	VAS, AR
Pelletier JP	2000	24 weeks	D (50, 100, 150)	Placebo	62.8	76	31.28	7.9	VAS
Reginster JY	2001	3 years	GS (1,500)	Placebo	65.8	79.4	27.35	7.8	WOMAC, JSW, AR
Dougados M	2001	3 years	D (100)	Placebo	62.6	84	-	-	Lequesne, JSW
Hughes R	2002	6 months	GS (1,500)	Placebo	62.3	68	-	7.63	AR
Pavelka K	2002	1, 2, 3 years	GS (1,500)	Placebo	62.4	78.5	25.7	10.55	Lequesne, JSW, AR
Braham R	2003	3 months	GS (2,000)	Placebo	42.2	28.3	-	12.97	AR
Cibere J	2004	24 weeks	GS (1,500)	Placebo	64.5	57.7	25.26	1.6	AR
McAlindon T	2004	12 weeks	GS (1,500)	Placebo	-	64	32.57	-	WOMAC, AR
Pham T	2004	1 year	D (100)	Placebo	64.7	65.3	29.6	-	Lequesne
Clegg DO	2006	24 weeks	GS (1,500)	Placebo, NSAIDs	58.7	42.2	31.73	10	WOMAC, AR
Zheng WJ	2006	12, 16 weeks	D (100)	NSAIDs	56.2	66.2	26.13	7.23	VAS, WOMAC, AR
Herrero-Beaumont G	2007	24 weeks	GS (1,500)	Placebo	63.9	88	27.65	7.31	Lequesne, AR
Louthrenoo W	2007	12, 24 weeks	D (50)	NSAIDs	54	73	26.85	3.58	WOMAC
Pavelka K	2007	12, 24 weeks	D (50)	Placebo	63.7	65.5	28.9	6.497	WOMAC, AR
Frestedt JL	2008	12 weeks	GS (1,500)	Placebo	59.1	60	32.24	-	WOMAC, AR
Kawasaki T	2008	18 weeks	GS (1,500)	Placebo	68.9	-	23.95	-	JSW
Rozendaal RM	2008	3, 12, 24 months	GS (1,500)	Placebo	63.4	69.4	27.95	-	JSW, AR
Sawitzke AD	2008	2 months	GS (1,500)	Placebo, NSAIDs	50.6	55.7	-	8.52	JSW
Brahmachari B	2009	12 weeks	D (50)	Placebo	49.1	83.6	24.66	2.76	VAS, WOMAC, AR
Madhu K	2013	6 weeks	GS (1,500)	Placebo	56.8	70	27.9	-	VAS, AR
Chopra A	2013	24 weeks	GS (1,500)	NSAIDs	55.5	-	27.7	-	WOMAC, AR
Durmus D	2013	12 weeks	GS (1,500)	Placebo	55.8	-	27.7	-	WOMAC
Kwoh CK	2014	24 weeks	GS (1,500)	Placebo	52.24	48.9	28.9	-	WOMAC, AR

AR = adverse event, BMI = body mass index, D = diacerein, GH = glucosamine hydrochloride, GS = glucosamine sulfate, JSW = joint space width, VAS = visual analog score.

Among 23 glucosamine studies [26-44,1-4], the comparators included placebo, NSAIDs, and both placebo and NSAIDS in 17 studies [39,30,29,33,43,31,42,41,32,38,26,27,35,37,4,1,2], 4 studies [40,36,34,48,28], and 2 studies [28,44], respectively. All studies used glucosamine sulfate, except for one study [31] which used glucosamine hydrochloride. Among eight diacerein studies, five studies [21,23,19,22,18], two studies [25,20], and 1 study [21] had comparators as placebo, NSAIDs, and both NSAIDs and placebo, respectively. Most studies (24/27) included OA of the knee and the rest were OA of the hip. Mean age, body mass index (BMI), and duration of OA varied from 42 to 69 years, 24.0 to 32.6 kg/m^2^, and 1.6 to 13 years, respectively. Percentage of females in each study ranged from 5.1% to 88%. Duration of treatment ranged from 4 weeks to 3 years. Various outcomes were compared between the treatment groups (Figure 1).

### Risk of bias in included studies

Risk of bias assessment is described in Additional file 2.

### Direct comparisons

Data used for direct comparisons for all treatments and outcomes were measured at the end of each study, as described in Table 1. Pooling according to outcomes was performed if there were at least two studies for each comparison, as clearly described below.

#### Visual analog score

Among eight studies [25,39,34,21,23,40,42,47], five studies compared glucosamine pain VAS with comparators of placebo [39,42,2] and NSAIDs [34,40], respectively. Three studies compared diacerein with placebo [21,23] and NSAIDs [25]. Most studies assessed pain using the VAS at 4 weeks to 3 years. Data for the mean and SD of VAS scores are described in Additional file 3. The mean VAS was −0.90 (95% CI: −1.67, −0.14) units significantly lower in glucosamine than in NSAIDs (Table 2). The mean VAS score was about −1.44 (95% CI: −3.01, 0.12) units lower in glucosamine than in placebo, but this was not significant. The pooled effects of diacerein *versus* placebo from three studies (*n* = 103 *vs.* 98) displayed no heterogeneity (*I*^2^ = 0%) with an UMD of −2.23 (95% CI: −2.82, −1.64). The effect of diacerein *versus* NSAIDs was not statistically different with an UMD of 0.149 (95% CI: −0.29, 0.59). There was no evidence of publication bias for both pooled effect estimates.

**Table 2:** **Summarized results of direct comparisons according to type of interventions**

**Clinical outcomes**	**No. studies**	***I*** **2**	**No. subjects**	**UMD (95% CI)**
VAS				
Glu *vs.* Pla	3	83.4	89 *vs.* 89	−1.44 (−3.01, 0.12)
Glu *vs.* NSAIDs	2	0	106 *vs.* 110	−0.90 (−1.67, −0.14)*
Dia *vs.* Pla	2	0	103 *vs.* 98	−2.23 (−2.82, −1.64)*
Dia *vs.* NSAIDs	2	0	181 *vs.* 182	0.15 (−0.29, 0.59)
Total WOMAC score change				
Glu *vs.* Pla	6	0	437 *vs.* 423	−2.49 (−4.14, −0.83)*
Actual score				
Glu *vs.* Pla	2	79.8	73 *vs.* 76	5.67 (−11.26, 22.61)
Pain WOMAC score change				
Glu *vs.* Pla	10	66.3	1,069 *vs.* 1,056	−0.75 (−1.18, −0.32)*
Glu *vs.* NSAIDs	2	85.5	425 *vs.* 423	−0.07 (−1.5, 1.36)
Actual score				
Glu *vs.* Pla	4	81	408 *vs.* 418	0.06 (−1.33, 1.45)
Functional WOMAC score change				
Glu *vs*. Pla	10	67.6	1,069 *vs.* 1,056	−0.58 (−1.98, 0.81)
Glu *vs.* NSAIDs	2	77.1	425 *vs.* 423	−0.84 (−2.95, 4.63)
Actual score				
Glu *vs.* Pla	4	90.5	408 *vs.* 418	−4.78 (−5.96, −3.59)*
Dia *vs.* Pla	2	0	110 *vs.* 110	−7.72 (−18.83, 3.39)
Dia *vs.* NSAIDs	2	92.9	189 *vs.* 185	−6.64 (−10.50, −2.78)*
Stiffness WOMAC score change				
Glu *vs.* Pla	7	68.1	759 *vs.* 743	−0.02 (−0.06, 0.03)
Actual score				
Glu *vs.* Pla	3	31.5	390 *vs.* 389	0.09 (−0.38, 0.56)
Dia *vs.* Pla	2	1	110 *vs.* 110	−0.68 ( −1.20, −0.16)^*^
Lequesne score scores change				
Glu *vs.* Pla	2	87.7	207 *vs.* 205	−1.03 (−1.34, −0.72) ^*^
Dia *vs.* Pla	2	0	340 *vs.* 337	0.002 (−0.704, 0.708)
Joint space width				
Glu *vs*. Pla	4	86.4	357 *vs.* 350	0.008 (−0.232, 0.248)
All adverse events				RR (95% CI)
Glu *vs.* Pla	16	7.3	1,366 *vs.* 1,365	1.12 (1.02, 1.23)*
Glu *vs.* NSAIDs	5	81.9	631 *vs.* 632	0.53 (0.24, 1.20)
Dia *vs.* Pla	4	76.6	275 *vs.* 278	5.58 ( 2.14, 14.59)*
Dia *vs.* NSAIDs	2	97.6	181 *vs.* 182	1.59 ( 0.47, 5.44)
GI adverse event				
Glu *vs.* Pla	14	0	1,217 *vs.* 1,211	0.99 ( 0.82, 1.19)
Glu *vs.* NSAIDs	5	74.3	631 *vs.* 632	0.51 ( 0.22, 1.20)
Dia *vs.* Pla	6	94.9	937 *vs.* 701	2.00 ( 0.69, 5.74)
Dia *vs.* NSAIDs	3	86.6	373 *vs.* 267	1.37 ( 0.89, 2.10)

*Statistically significant difference (*P* < 0.05). CI = confidence interval, NSAIDs = non-steroidal anti-inflammatory drugs, RR = relative risk, UMD = unstandardized mean difference, VAS = visual analog score, WOMAC = Western Ontario and McMaster Universities Osteoarthritis index.

#### WOMAC score

As described in Table 2, the total WOMAC scores were compared as change from baseline and the actual scores measured at the end of each study. Among six studies [38,27,35,30,29,33] with total WOMAC score changes, the effects displayed no heterogeneity (*I*^2^ = 0%) with an UMD of −2.49 (95% CI: −4.14, −0.83). The actual total WOMAC scores from two studies [31,29] were not statistically significant with an UMD of 5.67 (95% CI: −11.26, 22.61). The actual total WOMAC scores and change in sub-WOMAC scores (pain, stiffness, and function) were also compared (Table 2). Compared with placebo, glucosamine resulted in a significantly greater change in WOMAC pain scores with an UMD of −0.75 (95% CI: −1.18, −0.32). In addition, mean functional and stiffness WOMAC scores were significantly lower in the diacerein groups when compared to the NSAIDs and placebo groups (6.64 (95% CI: −10.50, −2.78) and −0.68 (95% CI: −1.20, −0.16)).

#### Lequesne score and joint space width

Only glucosamine resulted in a significantly greater change of Lequesne score when compared to placebo (UMD = −1.030 (95% CI: −1.34, −0.72)) (Table 2). However, glucosamine did not result in a significant change in joint space when compared to placebo with an UMD of 0.008 (95% CI: −0.232, 0.248).

#### Adverse events

Compared with a placebo control, composite adverse events were 1.12 (95% CI: 1.02, 1.23) and 5.58 (95% CI: 2.14, 14.59) times significantly higher in glucosamine and diacerein than in placebo (Table 2). When considering only GI adverse events, the pooled RR of glucosamine was 0.99 (95% CI: 0.82, 1.19) when compared with placebo and 0.393 (95% CI: 0.157, 0.588) when compared with NSAIDs. Conversely, diacerein respectively had 2.00 (95% CI: 0.69, 5.74) and 1.37 (95% CI: 0.89, 2.10) times more GI effects than placebo and NSAIDs, but this data was not statistically significant.

### Network meta-analysis

#### Visual analog score

Data from eight studies [39,34,40,42,21,23,25,2] were included in pooling of indirect comparisons of the VAS scores (Additional file 4). Mean VAS scores measured at 4 to 24 weeks after receiving treatments were fitted as the dependent variable in a mixed linear regression model.

**Figure 2:**
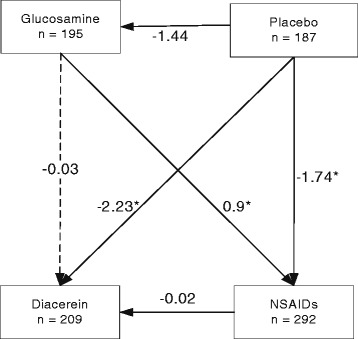
**Network meta-analysis of treatment effect on VAS.**

The VAS score was lowest in the diacerein group with an overall mean of 3.28 (95% CI: 2.25, 4.30) followed by the glucosamine (3.30, 95% CI: 2.61, 4.01), NSAIDs (3.31, 95% CI: 2.13, 4.50), and placebo groups (5.05, 95% CI: 3.79, 6.32). The regression analysis suggested that all active treatments resulted in a significant difference in VAS score when compared to placebo (Table 3). Multiple comparisons suggested no difference in effects between active treatments (Figure 2).

**Table 3:** **Comparisons of treatment effects: a network meta-analysis**

**Treatment**	***N***	***Β***	***P*** **value**	**95% CI**
Pain VAS score				
Glucosamine	195	−1.75	0.034*	−3.32, −0.17
NSAIDs	292	−1.74	0.047*	−3.44, −0.03
Diacerein	209	−1.78	0.024*	−3.24, −0.32
Placebo	187	0	-	-
Diacerein *vs.* glucosamine	-	−0.03	0.964	−1.58, 1.52
NSAIDs *vs.* glucosamine	-	0.01	0.988	−1.52, 1.54
Diacerein *vs.* NSAIDs	-	−0.04	0.893	−0.72, 0.64
Total WOMAC score				
Glucosamine	73	17.70	0.367	−35.46, 70.87
NSAIDs	79	−7.03	0.334	−26.51, 12.45
Diacerein	164	−6.2	0.597	−39.69, 27.29
Placebo	159	0	-	-
Diacerein *vs.* glucosamine	-	−23.90	0.218	−72.85, 25.06
NSAIDs *vs.* glucosamine	-	−24.74	0.180	−69.91, 20.44
Diacerein *vs.* NSAIDs	-	0.84	0.899	−18.52, 20.20
Pain WOMAC score				
Glucosamine	408	1.47	0.139	−0.74, 3.68
NSAIDs	164	−0.38	0.612	−2.31, 1.55
Diacerein	397	−0.75	0.521	−3.75, 2.24
Placebo	491	0	-	-
Diacerein *vs.* glucosamine	-	−2.22	0.078	−4.84, 0.39
NSAIDs *vs.* glucosamine	-	−1.85	0.065	−3.88, 0.18
Diacerein *vs.* NSAIDs	-	−0.376	0.643	−2.46, 1.71
Function WOMAC score				
Glucosamine	408	−2.42	0.534	−11.18, 6.33
NSAIDs	503	1.53	0.463	−3.13, 6.20
Diacerein	189	1.89	0.392	−3.02, 6.81
Placebo	407	0	-	-
Diacerein *vs.* glucosamine	-	4.32	0.436	−8.03, 16.67
NSAIDs *vs.* glucosamine	-	3.96	0.438	−7.43, 15.34
Diacerein *vs.* NSAIDs	-	0.36	0.913	−7.16, 7.89
Stiffness WOMAC score				
Glucosamine	390	−0.15	0.480	−0.66, 0.36
NSAIDs	397	−0.77	0.207	−2.14, 0.60
Diacerein	196	−0.89	0.097	−2.01, 0.23
Placebo	499	0	**-**	**-**
Diacerein *vs.* glucosamine	-	−0.74	0.193	2.00, −0.52
NSAIDs *vs.* glucosamine	-	0.62	0.205	−1.71, 0.47
Diacerein *vs.* NSAIDs	-	−0.12	0.788	−1.20, 0.96
Lequesne score change				
Glucosamine	214	−1.12	0.063	−2.36, 0.12
Diacerein	75	0.03	0.970	−2.13, 2.18
Placebo	192	0	-	-
Diacerein *vs.* glucosamine	**-**	1.15	0.331	−4.32, 2.01
Joint space width change				
Glucosamine	357	−0.08	0.363	−0.31, 0.14
Diacerein	246	0.12	0.207	−0.10, 0.34
Placebo	597	0	**-**	**-**
Diacerein *vs.* glucosamine	-	−0.20	0.001*	−0.27, −0.14
Adverse event	*N*	RR	*P* value	95% CI
Glucosamine	1,474	1.07	0.398	0.92, 1.23
NSAIDs	814	2.20	<0.001*	1.56, 3.11
Diacerein	381	1.91	<0.001*	1.36, 2.69
Placebo	1,634	1	-	-
Diacerein *vs.* glucosamine	-	1.80	0.001*	1.27, 2.55
NSAIDs *vs.* glucosamine	-	2.07	<0.001*	1.47, 2.91
Diacerein *vs.* NSAIDs	-	0.87	0.416	0.62, 1.22
Adverse event (GI)				
Glucosamine	1,850	0.84	0.103	0.68, 1.04
NSAIDs	899	1.33	0.038*	1.02, 1.77
Diacerein	1,345	1.44	<0.001*	1.24, 1.68
Placebo	1,917	1	-	-
Diacerein *vs.* glucosamine	-	1.72	<0.001*	1.34, 2.22
NSAIDs *vs.* glucosamine	-	1.60	0.004*	1.16, 2.19
Diacerein *vs.* NSAIDs	-	1.08	0.576	0.98, 1.50

*Statistically significant difference (*P* < 0.05). CI = confidence interval, GI = gastrointestinal, NSAIDs = non-steroidal anti-inflammatory drugs, RR = relative risk, VAS = visual analog score, WOMAC = Western Ontario and McMaster Universities Osteoarthritis index.

#### Total WOMAC score

Data from four studies [31,29,20,22] were included in pooling of indirect comparisons of the actual total WOMAC scores (Additional file 4). The mean total WOMAC scores were lower in the diacerein and NSAID groups, but higher in glucosamine compared to placebo, but this was not statistically significant (Table 3). Multiple comparisons suggested no difference in effects between active treatments.

#### Pain WOMAC score

Data from six studies [31,28,29,20,22,4] were included in the network meta-analysis of pain WOMAC scores (Additional file 4). Fitting the regression analysis using placebo as the reference suggested that pain WOMAC scores were lower in both the diacerein and NSAIDs groups. In contrast, the pain score was higher in the glucosamine group compared with placebo, but this was not statistically significant (Table 3).

#### Function WOMAC score

Data from eight studies [20,22,18,31,28,29,4,25] were included in pooling of indirect comparisons of WOMAC function scores (Additional file 4). The regression analysis suggested that mean WOMAC function scores of diacerein, NSAIDs, and glucosamine were lower than placebo, but these results were not statistically significant (Table 3). Multiple comparisons indicated that diacerein and NSAIDs resulted in lower scores than glucosamine, but these results also were not statistically significant.

#### Stiffness WOMAC score

Data from six studies [20,22,18,31,28,29] were included in pooling of indirect comparisons of the WOMAC stiffness scores (Additional file 4). The regression analysis suggested that mean WOMAC stiffness scores were lower in diacerein, NSAIDs, and glucosamine groups when compared to placebo (Table 3). There was no significant difference between the three active treatments.

#### Lequesne algofunctional score change

Three studies [21,36,37] compared mean changes of Lequesne scores after receiving treatments at 4 to 24 weeks (Additional file 4). The regression analysis suggested that mean Lequesne change in the glucosamine group was lower than the placebo group. There was no significant difference between the glucosamine and diacerein groups.

#### Joint space width difference

Data from five studies [33,38,43,41,19] were used for the network meta-analysis of joint space width change. Change of joint space width after receiving glucosamine and diacerein had no statistically significant difference when compared to placebo (Table 3). Multiple comparisons indicated that diacerein was superior to glucosamine at −0.2 mm (95% CI: −0.27, −0.14).

#### Adverse events

**Figure 3:**
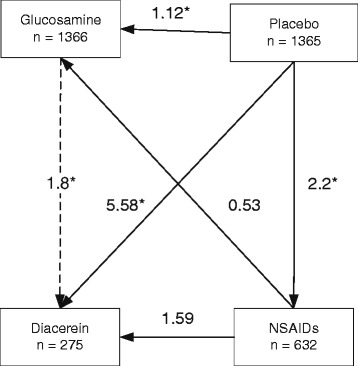
**Network meta-analysis of treatment effect on drug adverse effect.**

Sixteen studies [39,37,31,42,41,32,38,26,27,35,28,30,29,43,47,48] reported overall adverse events between treatment groups (Additional file 5). Compared to glucosamine, NSAIDs was 2.07 (95% CI: 1.47, 2.91) times and diacerein was 1.80 (95% CI: 1.27, 2.55) times more likely to have adverse events (Figure 3). Diacerein had approximately 13% (RR = 0.87; 95% CI: 0.62, 1.22) lower risk than NSAIDs, but this was not statistically significant (Table 3). Considering only GI adverse events showed similar results to overall adverse events.

## Discussion

This review compared effects of glucosamine, diacerein, NSAIDs, and placebo for the treatment of osteoarthritis of the knee. Relevant clinical outcomes that were pooled included VAS pain score, total and sub-WOMAC scores, joint space width, and adverse events.

The second-line drugs for OA knee in the SYSADOA group include glucosamine sulfate, glucosamine hydrochloride, chondroitin sulfate, hyaluronic acid, ASU, and diacerein. This study included both glucosamine sulfate and glucosamine hydrochloride because the results of previous meta-analysis of both preparations show that they have no statistically significant difference [49]. Chondroitin sulfate [50-52], hyaluronic acid [53], and ASU [54] were not included in this review because there were too few studies to pooled outcomes with network meta-analysis.

The clinical results of our study were consistent to previous meta-analyses [6,4,5,3] in which glucosamine and diacerein statistically improved pain scores (VAS and WOMAC) and function scores (WOMAC) when compared to placebo. However, we have added more evidence of multiple active treatment comparisons. There were no statistically significant differences between the three groups in pain VAS, total WOMAC, sub-WOMAC scores, and Lequesne functional scores. Although glucosamine showed greater improvement in joint space width when compared to diacerein, glucosamine and diacerein did not show a clinically relevant effect in joint space narrowing when compared to placebo. Both glucosamine and diacerein increased risk of adverse events when compared with placebo. However, glucosamine had a lower risk of adverse events when compared to diacerein. In the subgroup of gastrointestinal adverse events, patients who took diacerein had an approximately 86.9% and 99.6% increased risk of GI adverse events when compared to glucosamine and placebo, respectively. Glucosamine and diacerein can reduce pain VAS and improve function (WOMAC). Glucosamine and diacerein showed no differences in adverse effects when compared to NSAIDs. In conclusion, this study demonstrated that either glucosamine or diacerein can be selected for the treatment of pain associated with knee osteoarthritis. Diacerein has a higher risk of adverse GI events when compared to glucosamine. Both glucosamine and diacerein cannot decrease risk of adverse effects, and they both do not have a clinically relevant effect in delaying progression of joint space narrowing in OA of the knee.

The small number of studies that evaluated each particular pair of treatments limits performing a direct meta-analysis. A network meta-analysis circumvents this problem by creating indirect comparisons between active treatments that can identify the most effective therapy. In this case, diacerein was the best therapy for improvement of pain VAS scores. Glucosamine is the best therapy in terms of having less adverse effects when compared to diacerein but not when compared to placebo controls. None of the RCTs had compared combined treatments with an active control.

The strengths of this study were that a network meta-analysis was applied to increase the power of the tests and reduce type I errors [14-16]. We applied a regression model taking into account study effects to assess treatment effects. The network meta-analysis “borrows” treatment information from other studies and increases the total sample size. As a result, treatment effects that could not be detected in direct meta-analysis could be identified. All possible treatment comparisons are mapped and displayed (Additional file 6). Although our pooled estimates were heterogeneous, the regression model with cluster effect takes into account variations at the study level.

None of RCTs compared dual therapy with monotherapy of SYSADOA. In relation to the SYSADOA mechanism, diacerein inhibits IL-1b effects and reduces synthesis of cartilage-specific macromolecules. In addition, diacerein also decreases IL-1b-stimulated secretion of metalloproteinases and aggrecanases, thereby preventing breakdown of cartilage by these enzymes [55]. Glucosamine, an amino sugar, is a building block of the glycosaminoglycan, which is a part of the cartilage structure. The SYSADOA group should be able to support effects of each other and may yield better clinical improvement than monotherapy. Further RCTs that compare dual *vs*. monotherapy SYSADOA are necessary to determine if this may enhance treatment effects.

## Conclusions

This investigation demonstrates the potency of diacerein and glucosamine in the treatment of osteoarthritis of the knee. Glucosamine shows significant improvements in pain score but does not decrease risk of adverse effects and does not have a clinically relevant effect in slowing progression of joint space narrowing in OA knee. Diacerein has a higher risk of adverse GI events when compared to glucosamine. Diacerein also does not decrease risk of adverse effects and has no clinically relevant effect in delaying progression of joint space narrowing in OA of the knee. When compared to diacerein, glucosamine is the better treatment choice for OA of the knee.

## Additional files

Additional file 1:
**Search Strategies.**
Additional file 2: Table S2. Risk of bias assessment. Additional file 3: Table S3. Direct comparison of means VAS, WOMAC (total, pain, stiffness, and function), Lequesne actual, and difference score according to treatment. Additional file 4: Table S4. Sample size, mean, and SD between treatment groups for studies included in a network meta-analysis. Additional file 5: Table S5. Frequency of overall adverse events between treatment groups. Additional file 6: Table S6. Summarization all treatment effects for osteoarthritis patients.

## Abbreviations

ASU: avocado soybean unsaponifiables
BMI: body mass index
CI: confidence intervals
GI: gastrointestinal
NSAIDs: non-steroidal anti-inflammatory drugs
OA: osteoarthritis
RCTs: randomized controlled trials
RR: relative risk
SD: standard deviation
SYSADOA: symptomatic slow-acting drugs for OA
UMD: unstandardized mean difference
VAS: visual analog scores
WOMAC: Western Ontario and McMaster Universities Osteoarthritis index
