# Supplementary material for: Efficacy and safety of glucosamine, diacerein, and NSAIDs in osteoarthritis knee: a systematic review and network meta-analysis
Source: Eur J Med Res. 2015 Mar 13;20(1):24. doi: 10.1186/s40001-015-0115-7 (PMC4359794; doi:10.1186/s40001-015-0115-7)
Supplement: Additional file 1: — Search Strategies. [file 40001_2015_115_MOESM1_ESM.doc]

**Additional file 1: Appendix.** Search Strategies

#1 Osteoarthritis or Degenerative arthritis

#2 adult

#3 elderly

#4 #1 AND #2 AND #3

#5 “SYmptomatic Slow Acting Drug for OsteoArthritis”

#6 SYSADOA

#7 glucosamine

#8 diacerein

#9 #5 OR #6 OR #7 OR #8

#10 Pain

#11 Functions

#12 score

#13 grade

#14 WOMAC

#15 KSS

#16 motion

#17 radiographic grading

#18 x-ray

#19 MRI

#20 KellgrenLawrance

#21 #10 OR #11 OR #12 OR #13 OR #14 OR #15 OR #16 OR #17 OR #18 OR #19 OR #20

#22 clinical trial OR RCT OR randomize controlled trial

#23 #4 AND #9 AND #21 AND # 22
