# Supplementary material for: Efficacy and safety of glucosamine, diacerein, and NSAIDs in osteoarthritis knee: a systematic review and network meta-analysis
Source: Eur J Med Res. 2015 Mar 13;20(1):24. doi: 10.1186/s40001-015-0115-7 (PMC4359794; doi:10.1186/s40001-015-0115-7)
Supplement: Additional file 2: Table S2. — Risk of bias assessment. [file 40001_2015_115_MOESM2_ESM.doc]

**Additional file 2: Table S2. Risk of bias assessment**

| Author | Sequence generation | Allocation concealment | Blinding | Incomplete  outcome  data | Selective  outcome  report | Other source of  bias | Description of  other bias |
| --- | --- | --- | --- | --- | --- | --- | --- |
| Pujalte JM | U | U | Y | U | Y | U | Did not mention about ITT |
| Lopes VA | U | N | N | U | Y | U | Did not mention about ITT |
| Muller FH | U | U | Y | Y | Y | Y |  |
| Noack W | U | U | Y | Y | Y | Y |  |
| Nguyen M | U | U | Y | Y | Y | N | Unbalance between baseline age and disease duration |
| Qiu GX | U | U | Y | Y | Y | U | Did not mention about ITT |
| Houpt JB | Y | Y | Y | Y | Y | N | Unbalance between baseline WOMAC score |
| Rindone JP | U | U | Y | U | Y | U | Did not mention about ITT |
| Pelletier JP | Y | Y | Y | N | Y | Y |  |
| Reginster JY | Y | Y | Y | N | N | Y |  |
| Dougados M | Y | Y | Y | N | Y | Y |  |
| Hughes R | Y | Y | Y | Y | Y | U | Did not mention about ITT |
| Pavelka K | Y | Y | Y | N | Y | Y |  |
| Braham R | N | N | Y | Y | Y | Y |  |
| Cibere J | Y | Y | Y | Y | Y | Y |  |
| McAlindon T | U | Y | Y | Y | Y | Y |  |
| Pham T | Y | Y | Y | Y | Y | Y |  |
| Clegg DO | Y | Y | Y | Y | Y | Y |  |
| Zheng WJ | U | N | Y | Y | Y | U | Did not mention about ITT |
| Herrero-Beaumont G | Y | Y | Y | N | Y | Y |  |
| Louthrenoo W | U | N | Y | Y | Y | Y |  |
| Pavelka K | U | N | Y | Y | Y | Y |  |
| Frestedt JL | U | N | Y | N | Y | Y |  |
| Kawasaki T | U | N | N | N | Y | Y |  |
| Rozendaal RM | Y | N | Y | Y | Y | Y |  |
| Sawitzke AD | Y | Y | Y | N | Y | Y |  |
| Brahmachari B | U | N | N | Y | Y | Y |  |
| Madhu K | Y | Y | Y | Y | Y | Y |  |
| Chopra A | Y | Y | N | Y | Y | Y |  |
| Durmus D | Y | Y | N | N | Y | U | Did not mention about ITT |
| Kwoh CK | Y | Y | Y | Y | Y | Y |  |
