# Supplementary material for: Efficacy and safety of glucosamine, diacerein, and NSAIDs in osteoarthritis knee: a systematic review and network meta-analysis
Source: Eur J Med Res. 2015 Mar 13;20(1):24. doi: 10.1186/s40001-015-0115-7 (PMC4359794; doi:10.1186/s40001-015-0115-7)
Supplement: Additional file 3: Table S3. — Direct comparison of means VAS, WOMAC (total, pain, stiffness, and function), Lequesne actual, and difference score according to treatment. [file 40001_2015_115_MOESM3_ESM.doc]

**Additional file 3: Table S3.** Direct comparison of means VAS, WOMAC (total, pain, stiffness and function), Lequesne actual and difference score according to treatment.

| **Author** | | **Outcome ^a^** | **Active treatment** | | | | **Comparator** | | | |
| --- | --- | --- | --- | --- | --- | --- | --- | --- | --- | --- |
|  | |  | **Treatment** | | | | **NSAIDs** | | **Placebo** | |
|  | |  | **No. of participant** | | | **Score, mean(SD)** | **No. of participant** | **Score, mean(SD)** | **No. of participant** | **Score, mean(SD)** |
| **Pain score** | | | | | | | | | | |
| **Glucosamine** |  | | |  | |  |  |  |  |  |
| Pujalte JM, (1980) | VAS | | | 10 | | 3.13 (0.63) |  |  | 10 | 5.9 (1.98) |
| Rindone JP, (2000) | VAS | | | 49 | | 4.9 (2.8) |  |  | 49 | 4.9 (2.2) |
| Mudhu K, (2013) | VAS | | | 30 | | 2.93 (2.06) |  |  | 30 | 4.60 (2.08) |
| UMD (95%CI) |  | | | -1.44 (-3.01, 0.12) | | | | | | |
| Lopes VA, (1982) | VAS | | | 18 | | 2.7 (1.6) | 20 | 4 (2) |  |  |
| Qiu GX(1998 | VAS | | | 88 | | 3.6(4.07) | 90 | 4.18(2.81) |  |  |
| UMD (95%CI) |  | | | -0.90 (-1.67, -0.14 )* | | | | | |  |
| **Diacerein** |  | | |  | |  |  |  |  |  |
| Brahmachari B (2009) | VAS | | | 28 | | 3.39 (1.193) |  |  | 27 | 6.03(1.3) |
| Nguyen M, (1994) | VAS | | | 75 | | 4.0 (2.6) | 75 | 3.8 (2.7) | 71 | 4.8 (2.4) |
| UMD (95%CI) |  | | | -2.23 (-2.82, -1.64)* | | | | | |  |
| Zheng WJ, (2006) | VAS | | | 106 | | 2.86 (1.97) | 107 | 2.73 (1.83) |  |  |
| UMD (95%CI) | VAS | | | 0.15 (-0.29, 0.59) | | | | | | |
| **Glucosamine** |  | | |  | |  |  |  |  |  |
| Houpt JB, (1999) | Total WOMAC  Score | | | 58 | | 36.57 (19.50) |  |  | 60 | 38.57 (19.30) |
| Frestedt JL, (2008) | Total WOMAC score | | | 15 | | 70.2 (17.6) |  |  | 16 | 54.8 (22.7) |
| UMD (95%CI) |  | | | 5.67 (-11.26, 22.61) | | | | | |  |
| **Diacerein** |  | | |  | |  |  |  |  |  |
| Louthrenoo W, (2007) | Total WOMAC score | | | 82 | | 16.67 (17.76) | 79 | 35.86 (27.69) |  |  |
| Pavelka K, (2007) |  | | | 82 | | 30.36 (20.45) |  |  | 83 | 39.4 (21.87) |
| **Glucosamine** | **Score change** | | |  | |  |  |  |  |  |
| Pavelka K, (2002 | Total WOMAC change | | | 101 | | -8 (8.72) |  |  | 101 | -4.9 (8.72) |
| Cibere J, (2004) |  | | | 71 | | -3.24 (15.52) |  |  | 66 | -3.4 (18.12) |
| McAlindon T, (2004) |  | | | 101 | | 7.8 (13.1) |  |  | 104 | 7.8 (13.5) |
| Herrero-Beaumont G, (2007) |  | | | 106 | | -12.9 (14.71) |  |  | 104 | -8.2 (16.13) |
| Frestedt J, (2008) |  | | | 15 | | -10.9 (15.6) |  |  | 16 | -6.1 (17.6) |
| Kawasaki T, (2008) |  | | | 39 | | -14.9 (15.9) |  |  | 32 | -11.5 (13.2) |
| UMD (95%CI) |  | | | -2.49 (-4.14, -0.83)* | | | | | |  |
| **Glucosamine** |  | | |  | |  |  |  |  |  |
| Houpt JB, (1999) | Pain WOMAC score | | | 58 | | 7.14 (4.01) |  |  | 60 | 7.65 (4.13) |
| Clegg DO, (2006) |  | | | 317 | | 5.97 (4.64) | 318 | 5.43 (4.33) | 313 | 6.04 (4.52) |
| Frestedt JL, (2008) |  | | | 15 | | 14.58 (3.52) |  |  | 16 | 10.58 (4.28) |
| Durmus D (2013) |  | | | 18 | | 0.55 (0.7) |  |  | 19 | 1.94 (1.99) |
| UMD (95%CI) |  | | | 0.06 (-1.33, 1.45) | | | | | |  |
| **Diacerein** |  | | |  | |  |  |  |  |  |
| Louthrenoo W, (2007) | Pain WOMAC score | | | 82 | | 3.32 (3.53) | 79 | 8.05 (3.53) |  |  |
| Pavelka K, (2007) |  | | | 82 | | 4.36 (1.49) |  |  | 83 | 4.2 (1.6) |
| **Glucosamine** | **Score change** | | |  | |  |  |  |  |  |
| Pavelka K, (2002) | Pain WOMAC score | | | 101 | | -2.0 (2.56) |  |  | 101 | -1.3 (11.18) |
| Ringister JY, (2001) |  | | | 106 | | -1.35 (3.78) |  |  | 106 | -0.33 (4.46) |
| Cibere J, (2004) |  | | | 71 | | -1 (3.92) |  |  | 66 | -1.12 (4.16) |
| McAlindon T, (2004) |  | | | 101 | | 2 (3.4) |  |  | 104 | 2.5 (3.8) |
| Clegg D, (2006) |  | | | 317 | | -3.36 (4.62) | 318 | -4 (4.12) | 313 | -3.44 (4.57) |
| Herrero-Beaumont G, (2007) |  | | | 106 | | -2.7 (3.15) |  |  | 104 | -1.8 (3.64) |
| Frestedt J, (2008) |  | | | 15 | | -12.6 (16.3) |  |  | 16 | -2.9 (19.9) |
| Kawasaki T, (2008) |  | | | 39 | | -3.6 (4.4) |  |  | 32 | -2. (2.7) |
| Rozendaal R, (2008) |  | | | 111 | | -1. 9(1.6) |  |  | 111 | -0.3 (1.6) |
| Chopra A (2013) |  | | | 108 | | -2.72(3.29) | 105 | -1.9(3.03) |  |  |
| Kwoh CK (2014) |  | | | 98 | | -3.48(4.2) |  |  | 103 | -4.18(10.67) |
| UMD (95%CI) |  | | | -0.75(-1.18, -0.32)* | | | | | |  |
|  |  | | | -0.07 (-1.5, 1.36) | | | | | |  |
| **Glucosamine** | **Score change** | | |  |  | |  |  |  |  |
| Houpt JB, (1999) | Stiffness WOMAC score | | | 58 | 3.39 (1.81) | |  |  | 60 | 3.73 (1.76) |
| Clegg DO, (2006) |  | | | 317 | 2.82 (2.09) | | 318 | 2.67 (2.06) | 313 | 2.82 (1.95) |
| Frestedt JL, (2008) |  | | | 15 | 2.49 (0.79) | |  |  | 16 | 1.85 (1.01) |
| UMD (95%CI) |  | | | 0.091 (-0.381, 0.564) | | | | | |  |
| **Diacerein** |  | | |  | | | | | | |
| Louthrenoo W, (2007) |  | | | 86 | 1.44(1.49) | | 79 | 2.28 (2.25) |  |  |
| Pavelka K, (2007) |  | | | 82 | 2.68 (1.87) | |  |  | 83 | 3.52 (2.06) |
| Brahmachari B (2009) |  | | | 28 | 1.82 (1.35) | |  |  | 27 | 2.06 (2.31)) |
| UMD (95%CI) |  | | | -0.681 (-1.200, -0.162)* | | | | | |  |
| **Glucosamine** | **Score change** | | |  |  | |  |  |  |  |
| Pavelka K, (2002) | Stiffness WOMAC score | | | 101 | -0.31 (1.95) | |  |  | 101 | 0.11 (1.18) |
| Cibere J, (2004) |  | | | 71 | 0.08 (1.68) | |  |  | 66 | 0.24 (1.92) |
| McAlindon T, (2004) |  | | | 101 | 0.7 (1.6) | |  |  | 104 | 0.8 (1.5) |
| Clegg D, (2006) |  | | | 317 | -1.39 (2.1) | | 318 | -1.66 (2.41) | 313 | -1.46 (2.09) |
| Frestedt J, (2008) |  | | | 15 | -0.42 (0.96) | |  |  | 16 | -0.24 (0.73) |
| Kawasaki T, (2008) |  | | | **39** | -1.5 (1.7) | |  |  | 32 | -0.7 (1.3) |
| Rozendaal R, (2008) |  | | | 111 | -0.14(1.05) | |  |  | 111 | -0.09 (0.96) |
| UMD (95%CI) |  | | | -0.019 (-0.063, 0.026) | | | | | |  |
| **Glucosamine** |  | | |  |  | |  |  |  |  |
| Houpt JB, (1999) | Function WOMAC score | | | 58 | 25.98 (14.7) | |  |  | 60 | 27.17 (14.1) |
| Clegg DO, (2006) |  | | | 317 | 21.27 (15.54) | | 318 | 20 (15.31) | 313 | 21.61 (14.96) |
| Frestedt JL, (2008) |  | | | 15 | 48.08 (12.51) | |  |  | 15 | 38.35 (16.32) |
| Durmus (2013) |  | | | 18 | 2.94(2.91) | |  |  | 19 | 9.89 (1.13) |
| UMD (95%CI) |  | | | -4.78(-5.96, -3.59)* | | | | | |  |
| **Diacerein** |  | | |  |  | |  |  |  |  |
| Zheng WJ, (2006) | Function WOMAC score | | | 107 | 12.31 (9) | | 106 | 14.60 (9.84) |  |  |
| Louthrenoo W, (2007) |  | | | 82 | 11.91 (12.92) | | 79 | 25.56 (20.66) |  |  |
| UMD (95%CI) |  | | | -7.721 (-18.833, 3.391) | | | | | |  |
| Pavelka K, (2007) |  | | | 82 | 21.76 (14.64) | |  |  | 83 | 28.2 (15.68) |
| Brahmachari B (2009) |  | | | 28 | 24.51 (13.47) | |  |  | 27 | 31.6 (13.06) |
| UMD (95%CI) |  | | | -6.637 (-10.500, -2.775)* | | | | | |  |
| **Glucosamine** | **Score change** | | |  |  | |  |  |  |  |
| Pavelka K, (2002) | Function WOMAC score | | | 101 | -5.8 (7.18) | |  |  | 101 | -3.7 (6.15) |
| Ringister JY, (2001) |  | | | 106 | 6.53 (9.61) | |  |  | 106 | 2.67 (13.73) |
| Cibere J, (2004) |  | | | 71 | -2.32 (10.8) | |  |  | 66 | -2.52 (12.72) |
| McAlindon T, (2004) |  | | | 101 | 5.2 (9.5) | |  |  | 104 | 4.6 (9.6) |
| Clegg D, (2006) |  | | | 317 | -8.89 (15.53) | | 318 | -289.3 (340.7) | 313 | -9.09 (14.51) |
| Herrero-Beaumont G, (2007) |  | | | 106 | -9.2 (10.51) | |  |  | 104 | -5.5 (11.47) |
| Frestedt J, (2008) |  | | | 15 | -10.6 (15.4) | |  |  | 16 | -7 (18.4) |
| Kawasaki T, (2008) |  | | | 39 | -9.8 (11) | |  |  | 32 | -7.5 (9.8) |
| Rozendaal R, (2008) |  | | | 111 | -1.69 (1.3) | |  |  | 111 | 0.38 (1.3) |
| Chopra (2013) |  | | | 105 | -8.12(11.02) | | 108 | -6.93(10.04) |  |  |
|  |  | | | 98 |  | |  |  | 103 |  |
| UMD (95%CI) |  | | | -0.58 (-1.98, 0.81) | | | | | |  |
|  |  | | | -0.84 (-2.95, 4.63) | | | | | |  |
| **Glucosamine** |  | | |  |  | |  |  |  |  |
| Muller FH, (1994) | actual Lequesne score | | | 94 | 9.6 (5.82) | | 95 | 9.6 (5.85) |  |  |
| Noack W, (1994) | actual Lequesne score | | | 120 | 7.4 (5.48) | |  |  | 121 | 8.4 (4.4) |
| **Diacerein** |  | | |  |  | |  |  |  |  |
| Nguyen M, (1994) | actual Lequesne score | | | 75 | 7.7 (4.6) | | 75 | 6.9 (4.6) | 71 | 8.4 (4.1) |
| **Glucosamine** |  | | |  |  | |  |  |  |  |
| Pavelka K, (2002) | Lequesne score change | | | 101 | -1.7 (2.56) | |  |  | 101 | -0.82 (1.59) |
| Herrero-Beaumont G, (2007) | Lequesne score change ) | | | 106 | -3.1 (4.2) | |  |  | 104 | -1.9 (3.64) |
| UMD (95%CI) |  | | | -1.03 (-1.34, -0.72)* | | | | | |  |
| **Diacerein** |  | | |  |  | |  |  |  |  |
| Dougados M, (2001) | Lequesne score change | | | 255 | -0.5 (4) | |  |  | 252 | -0.5 (4.2) |
| Pham T, (2004) | Lequesne score change | | | 85 | -18.8 (14.7) | |  |  | 85 | -18.9 (16.9) |
| UMD (95%CI) |  | | | 0.002 (-0.704, 0.708) | | | | | |  |
| **Glucosamine** |  | | |  |  | |  |  |  |  |
| Ringister JY, (2001) | Joint space width | | | 106 | -0.06(0.79) | |  |  | 106 | 0.31 (0.95) |
| Pavelka K, (2002) | Joint space width | | | 101 | 0.04 (0.51) | |  |  | 101 | -0.19  (0.51) |
| Kawasaki T, (2008) | Joint space width | | | 39 | 0.0 (1.0) | |  |  | 32 | -0.3 (1.0) |
| Rozendaal R, (2008) | Joint space width | | | 111 | -0.094 (0.32) | |  |  | 111 | -0.057 (0.32) |
| UMD (95%CI) |  | | | 0.008 (-0.232, 0.248) | | | | | |  |
| **Diacerein** |  | | |  |  | |  |  |  |  |
| Dougados M, (2001) | Joint space width | | | 246 | 0.18 (0.25) | |  |  | 247 | 0.23 (0.23) |

^a^outcome score: VAS, total WOMAC, pain WOMAC, stiffness WOMAC, function WOMAC, actual Lequesne, Lequesne score change, joint space width

*statistically significant difference (P < 0.05)
