# Supplementary material for: Efficacy and safety of glucosamine, diacerein, and NSAIDs in osteoarthritis knee: a systematic review and network meta-analysis
Source: Eur J Med Res. 2015 Mar 13;20(1):24. doi: 10.1186/s40001-015-0115-7 (PMC4359794; doi:10.1186/s40001-015-0115-7)
Supplement: Additional file 4: Table S4. — Sample size, mean, and SD between treatment groups for studies included in a network meta-analysis. [file 40001_2015_115_MOESM4_ESM.doc]

**Additional file 4: Table S4.** Sample Size, Mean, and SD between treatment groups for studies included in a Network Meta-analysis.

| **Author** | **Treatments** | **VAS pain score** | | |
| --- | --- | --- | --- | --- |
|  |  | **N** | **Mean** | **SD** |
| Pujalte, JM, (1980) | Glucosamine sulfate  Placebo | 10 | 3.13 | 0.63 |
|  |  | 10 | 5.9 | 1.98 |
| Lopes VA, (1982) | Glucosamine sulfate  NSAIDs | 18 | 2.7 | 1.6 |
|  |  | 20 | 4 | 2 |
| Qiu GX, (1998) | Glucosamine sulfate  NSAIDs | 88 | 3.6 | 4.07 |
|  |  | 90 | 4.18 | 2.81 |
| Rindone JP, (2000) | Glucosamine sulfate | 49 | 4.9 | 2.8 |
|  | Placebo | 49 | 4.9 | 2.2 |
| Mudhu D (2013) | Glucosamine sulfate  Placebo | 30  30 | 2.93  4.60 | 2.06  2.08 |
| Brahmachari B, (2009) | Diacerein 50 mg  Placebo | 28 | 3.39 | 1.19 |
|  |  | 27 | 6.03 | 1.3 |
| Zheng WJ, (2006) | Diacerein 50 mg  NSAIDs | 106 | 2.86 | 1.97 |
|  |  | 107 | 2.73 | 1.83 |
| Nguyen M, (1994) | Diacerein 50 mg  NSAIDs  Placebo | 75 | 4 | 2.6 |
|  |  | 75 | 3.8 | 2.7 |
|  |  | 71 | 4.8 | 2.4 |
| **Author** | **Treatments** | **Total WOMACscore** | | |
|  |  | **N** | **Mean** | **SD** |
| Houpt JB, (1999) | Glucosamine sulfate | 58 | 36.57 | 19.50 |
|  | Placebo | 60 | 38.57 | 19.30 |
| Frestedt JL, (2008) | Glucosamine sulfate | 15 | 70.20 | 17.60 |
|  | Placebo | 16 | 54.8 | 22.7 |
| Louthrenoo W, (2007) | Diacerein 50 mg  NSAIDs | 82 | 16.67 | 17.76 |
|  |  | 79 | 35.86 | 27.69 |
| Pavelka K, (2007) | Diacerein 50 mg | 82 | 30.36 | 20.45 |
|  | Placebo | 83 | 39.4 | 21.87 |
| **Author** | **Treatments** | **Pain WOMAC score** | | |
|  |  | **N** | **Mean** | **SD** |
| Houpt JB, (1999) | Glucosamine sulfate | 58 | 7.14 | 4.01 |
|  | Placebo | 60 | 7.65 | 4.13 |
| Clegg DO, (2006) | Glucosamine sulfate | 317 | 5.97 | 4.64 |
|  | NSAIDs | 318 | 5.43 | 4.33 |
|  | Placebo | 313 | 6.04 | 4.52 |
| Frestedt JL, (2008 | Glucosamine sulfate | 15 | 14.58 | 3.52 |
|  | Placebo | 16 | 10.58 | 4.28 |
| Durmus D (2013) | Glucosamine sulfate | 18 | 0.55 | 0.7 |
|  | Placebo | 19 | 1.94 | 1.99 |
| Louthrenoo W, (2007) | Diacerein 50 mg | 86 | 3.32 | 3.53 |
|  | NSAIDs | 85 | 8.05 | 3.53 |
| Pavelka K, (2007) | Diacerein 50 mg | 82 | 4.36 | 1.49 |
|  | Placebo | 83 | 4.2 | 1.60 |
| **Author** | **Treatments** | **Stiffness WOMAC score** | | |
|  |  | **N** | **Mean** | **SD** |
| Houpt JP, (1999) | Glucosamine sulfate | 58 | 3.39 | 1.81 |
|  | Placebo | 60 | 3.73 | 1.76 |
| Clegg DO, (2006) | Glucosamine sulfate | 317 | 2.82 | 2.09 |
|  | NSAIDs | 318 | 2.67 | 2.06 |
|  | Placebo | 313 | 2.82 | 1.95 |
| Frestedt JL, (2008 | Glucosamine sulfate | 15 | 2.47 | 0.79 |
|  | Placebo | 16 | 1.85 | 1.01 |
| Louthrenoo W, (2007) | Diacerein 50 mg | 86 | 1.44 | 1.49 |
|  | NSAIDs | 85 | 2.28 | 2.25 |
| Pavelka K, (2007) | Diacerein 50 mg | 82 | 2.68 | 1.87 |
|  | Placebo | 83 | 3.52 | 2.06 |
| Brahmachari B, (2009) | Diacerein 50 mg | 28 | 1.82 | 1.35 |
|  | Placebo | 27 | 2.06 | 2.31 |
| **Author** | **Treatments** | **Function WOMAC score** | | |
|  |  | **N** | **Mean** | **SD** |
| Houpt JP, (1999) | Glucosamine sulfate | 58 | 25.98 | 14.7 |
|  | Placebo | 60 | 27.17 | 14.1 |
| Clegg DO, (2006) | Glucosamine sulfate | 317 | 21.27 | 15.54 |
|  | NSAIDs | 318 | 20.00 | 15.31 |
|  | Placebo | 313 | 21.61 | 14.96 |
| Frestedt JL, (2008) | Glucosamine sulfate | 15 | 48.08 | 12.51 |
|  | Placebo | 16 | 38.35 | 16.32 |
| Durmus D (2013) | Glucosamine sulfate  Placebo | 18  19 | 2.94  9.89 | 2.91  1.13 |
| Louthrenoo W, (2007) | Diacerein 50 mg | 82 | 11.91 | 12.92 |
|  | NSAIDs | 79 | 25.56 | 20.66 |
| Pavelka K, (2007) | Diacerein 50 mg | 82 | 21.76 | 14.64 |
|  | Placebo | 83 | 28.2 | 13.06 |
| Brahmachari B, (2009) | Diacerein 50 mg | 28 | 24.51 | 13.47 |
|  | Placebo | 27 | 31.6 | 13.06 |
| **Author** | **Treatments** | **Lequensealgofuctional change** | | |
|  |  | **N** | **Mean** | **SD** |
| Muller FH, (1994) | Glucosamine sulfate | 94 | 9.6 | 5.82 |
|  | NSAIDs | 95 | 9.6 | 5.85 |
| Noack W, (1994) | Glucosamine sulfate | 120 | 7.4 | 5.48 |
|  | Placebo | 121 | 8.4 | 4.4 |
| Nguyen M, (1994) | Diacerein 50 mg | 75 | 7.7 | 4.6 |
|  | NSAIDs | 75 | 6.9 | 4.6 |
|  | Placebo | 71 | 8.4 | 4.1 |
| **Author** | **Treatments** | **Joint space width change (mm)** | | |
|  |  | **N** | **Mean** | **SD** |
| Reginster JY, (2001) | Glucosamine sulfate | 106 | -0.06 | 0.79 |
|  | Placebo | 106 | 0.31 | 0.95 |
| Pavelka K, (2002) | Glucosamine sulfate | 101 | 0.04 | 0.1 |
|  | Placebo | 101 | -0.19 | 0.51 |
| Kawasaki T, (2008) | Glucosamine sulfate | 39 | 0 | 1 |
|  | Placebo | 32 | -0.3 | 1 |
| Rozendaal RM, (2008) | Glucosamine sulfate | 111 | -0.094 | 0.32 |
|  | Placebo | 111 | -0.057 | 0.32 |
| Dougados M, (2001) | Diacerein 50 mg | 246 | 0.18 | 0.25 |
|  | Placebo | 247 | 0.23 | 0.23 |
