# Supplementary material for: Efficacy and safety of glucosamine, diacerein, and NSAIDs in osteoarthritis knee: a systematic review and network meta-analysis
Source: Eur J Med Res. 2015 Mar 13;20(1):24. doi: 10.1186/s40001-015-0115-7 (PMC4359794; doi:10.1186/s40001-015-0115-7)
Supplement: Additional file 5: Table S5. — Frequency of overall adverse events between treatment groups. [file 40001_2015_115_MOESM5_ESM.doc]

**Additional file 5: Table S5**. Frequency of overall adverse events between Treatment groups

| **Author** | **Treatment** | **All adverse event** | | |
| --- | --- | --- | --- | --- |
|  |  | **N** | **Adverse event** | **No adverse event** |
| Pujalte JM, (1980) | Glucosamine sulfate | 10 | 0 | 10 |
|  | Placebo | 10 | 1 | 9 |
| Noack W, (1994) | Glucosamine sulfate | 126 | 8 | 118 |
|  | Placebo | 126 | 13 | 113 |
| Lopes VA, (1982) | Glucosamine sulfate | 18 | 2 | 16 |
|  | NSAIDs | 20 | 5 | 15 |
| Muller FH, (1994) | Glucosamine sulfate | 100 | 6 | 94 |
|  | NSAIDs | 99 | 35 | 64 |
| Qiu GX, (1998) | Glucosamine sulfate | 88 | 5 | 83 |
|  | NSAIDS | 90 | 14 | 76 |
| Houpt JB, (1999) | Glucosamine sulfate | 58 | 7 | 51 |
|  | Placebo | 60 | 7 | 53 |
| Rindone J, (2000) | Glucosamine sulfate | 49 | 2 | 47 |
|  | Placebo | 49 | 4 | 45 |
| Reginster JY, (2001) | Glucosamine sulfate | 106 | 101 | 5 |
|  | Placebo | 106 | 83 | 23 |
| Hughes R, (2002) | Glucosamine sulfate | 39 | 23 | 16 |
|  | Placebo | 39 | 25 | 14 |
| Pavelka K, (2002) | Glucosamine sulfate | 101 | 85 | 16 |
|  | Placebo | 101 | 69 | 32 |
| Braham R, (2003) | Glucosamine sulfate | 24 | 11 | 13 |
|  | Placebo | 22 | 10 | 12 |
| Cibere J, (2004) | Glucosamine sulfate | 71 | 0 | 71 |
|  | Placebo | 66 | 0 | 66 |
| McAlindon T, (2004) | Glucosamine sulfate | 101 | 18 | 83 |
|  | Placebo | 104 | 14 | 90 |
| Clegg DO, (2006) | Glucosamine sulfate | 317 | 9 | 308 |
|  | NSAIDs | 318 | 7 | 311 |
|  | Placebo | 313 | 11 | 302 |
| Herrero-Beaumont G, (2007) | Glucosamine sulfate | 106 | 44 | 62 |
|  | Placebo | 104 | 35 | 69 |
| Frestedt J, (2008) | Glucosamine sulfate | 19 | 12 | 7 |
|  | Placebo | 16 | 14 | 2 |
| Rozendaal R, (2008) | Glucosamine sulfate | 111 | 57 | 54 |
|  | Placebo | 111 | 59 | 55 |
| Chopra A (2013) | Glucosamine sulfate | 108 | 34 | 74 |
|  | NSAIDs | 105 | 34 | 71 |
| Mudhu K (2013) | Glucosamine sulfate | 30 | 5 | 25 |
|  | Placebo | 30 | 2 | 28 |
| Kwoh CK (2014) | Glucosamine sulfate | 98 | 3 | 95 |
|  | Placebo | 103 | 4 | 99 |
| Zheng WJ, (2006) | Diacerein 100 mg | 106 | 42 | 64 |
|  | NSAIDs | 107 | 50 | 57 |
| Pavelka K, (2007) | Diacerein 50 mg | 82 | 36 | 46 |
|  | Placebo | 83 | 24 | 59 |
| Brahmachari B, (2009) | Diacerein 50 mg | 28 | 27 | 1 |
|  | Placebo | 27 | 8 | 19 |
| Nguyen M, (1994) | Diacerein 50 mg | 75 | 68 | 7 |
|  | NSAIDs | 75 | 30 | 45 |
|  | Placebo | 71 | 33 | 38 |
| Author | **Treatment** | **GI adverse event** | | |
|  |  | **N** | **Adverse event** | **No adverse event** |
| Pujalte J, (1980) | Glucosamine sulfate | 10 | 0 | 10 |
|  | Placebo | 10 | 0 | 10 |
| Noack W, (1994) | Glucosamine sulfate | 126 | 5 | 121 |
|  | Placebo | 126 | 6 | 120 |
| Lopes VA. (1982) | Glucosamine sulfate | 18 | 1 | 17 |
|  | NSAIDs | 20 | 2 | 18 |
| Muller FH, (1994) | Glucosamine sulfate | 100 | 5 | 95 |
|  | NSAIDs | 99 | 29 | 70 |
| Qiu GX, (1998) | Glucosamine sulfate | 88 | 3 | 85 |
|  | NSAIDS | 90 | 6 | 84 |
| Houpt JB, (1999) | Glucosamine sulfate | 58 | 7 | 51 |
|  | Placebo | 60 | 7 | 53 |
| Reginster JY, (2001) | Glucosamine sulfate | 106 | 27 | 79 |
|  | Placebo | 106 | 37 | 69 |
| Hughes R, (2002) | Glucosamine sulfate | 39 | 0 | 39 |
|  | Placebo | 39 | 0 | 39 |
| Pavelka K, (2002) | Glucosamine sulfate | 101 | 25 | 76 |
|  | Placebo | 101 | 28 | 73 |
| Braham R, (2003) | Glucosamine sulfate | 24 | 4 | 20 |
|  | Placebo | 22 | 3 | 19 |
| Cibere J, (2004) | Glucosamine sulfate | 71 | 0 | 71 |
|  | Placebo | 66 | 0 | 66 |
| McAlindon T, (2004) | Glucosamine sulfate | 101 | 4 | 97 |
|  | Placebo | 104 | 6 | 98 |
| Clegg J, (2006) | Glucosamine sulfate | 317 | 0 | 317 |
|  | NSAIDs | 318 | 0 | 318 |
|  | Placebo | 313 | 0 | 313 |
| Herrero-Beaumont G, (2007) | Glucosamine sulfate | 106 | 11 | 95 |
|  | Placebo | 104 | 12 | 92 |
| Frestedt J(2008) | Glucosamine sulfate | 19 | 5 | 14 |
|  | Placebo | 16 | 6 | 10 |
| Rozendaal RM, (2008) | Glucosamine sulfate | 111 | 58 | 53 |
|  | Placebo | 111 | 46 | 65 |
| Mudhu K (2014) | Glucosamine sulfate | 30 | 1 | 29 |
|  | Placebo | 30 | 1 | 29 |
| Chopra A (2014) | Glucosamine sulfate | 108 | 34 | 74 |
|  | NSAIDs | 105 | 34 | 71 |
| Pelletier J, (2000) | Diacerein 100 mg | 385 | 192 | 193 |
|  | Placebo | 125 | 32 | 93 |
| Dougados M, (2001) | Diacerein 100 mg | 255 | 185 | 70 |
|  | Placebo | 252 | 115 | 137 |
| Pham T, (2004) | Diacerein 100 mg | 85 | 25 | 60 |
|  | Placebo | 85 | 72 | 13 |
| Zheng WJ, (2006) | Diacerein 100 mg | 106 | 29 | 77 |
|  | NSAIDs | 107 | 35 | 72 |
| Pavelka K, (2007) | Diacerein 50 mg | 82 | 25 | 57 |
|  | Placebo | 83 | 14 | 69 |
| Nguyen M, (1994) | Diacerein 50 mg | 75 | 60 | 15 |
|  | NSAIDs | 75 | 28 | 47 |
|  | Placebo | 71 | 24 | 47 |
