# Supplementary material for: Efficacy and safety of glucosamine, diacerein, and NSAIDs in osteoarthritis knee: a systematic review and network meta-analysis
Source: Eur J Med Res. 2015 Mar 13;20(1):24. doi: 10.1186/s40001-015-0115-7 (PMC4359794; doi:10.1186/s40001-015-0115-7)
Supplement: Additional file 6: Table S6. — Summarization all treatment effects for osteoarthritis patients. [file 40001_2015_115_MOESM6_ESM.doc]

**Additional file 6: Table S6**. Summarization all Treatment Effects for Osteoarthritis patients.

| **Treatments** | **Pain VAS score** | **WOMAC total score** | **WOMAC pain**  **score** | **WOMAC function score** | **WOMAC stiffness score** | **Lequesne mean difference** | **Joint space width** | **Adverse event** | **Adverse event (GI)** |
| --- | --- | --- | --- | --- | --- | --- | --- | --- | --- |
| Glu vs. Pla | N | D* | D* | D | X | D | X | D | X |
| NSAIDs vs. Pla | N | X | X | X | X | - | - | N | N |
| Dia vs. Plac | D&N | X | X | X | D | X | X | D&N | N |
| Glu vs. Dia | X | X | X | X | X | X | N | N | N |
| Glu vs. NSAIDs | D | X | X | X | X | - | - | N | N |
| Diac vs. NSAIDs | X | X | X | D | D | - | - | X | X |

D=direct, N=network

*Score change
